# Effectiveness of a Home-Based and Group-Based Tele-Exercise Program for Breast Cancer Survivors: Pilot Randomized Controlled Trial

**DOI:** 10.2196/79564

**Published:** 2026-06-26

**Authors:** Yang Gao, Yan Sun, Xueying Wang, Oi Kwan Chun, Brigitte Kim Yook Fung, Wai Wun Sara Fung

**Affiliations:** 1 Department of Sports and Health Sciences, Academy of Wellness and Human Development, Faculty of Arts and Social Sciences, Hong Kong Baptist University, AAB 935, Level 9, Academic and Administration Building, Baptist University Road Campus, Hong Kong, China (Hong Kong), 852 3411 7770; 2 Department of Surgery, Kwong Wah Hospital, Hong Kong, China (Hong Kong); 3 Department of Physiotherapy, Kwong Wah Hospital, Hong Kong, China (Hong Kong)

**Keywords:** breast cancer survivors, home-based and group-based tele-exercise program, randomized controlled trial, pilot study, telehealth, health-related quality of life

## Abstract

**Background:**

Breast cancer remains the most prevalent cancer among women globally. Adjuvant therapies can cause adverse effects that compromise physical and mental health. Exercise may mitigate these effects; however, many breast cancer survivors remain insufficiently active.

**Objective:**

This pilot study aimed to test the effectiveness of a 7-week theory-informed tele-exercise intervention for breast cancer survivors in Hong Kong.

**Methods:**

We developed a 12-week theory-informed tele-exercise intervention for breast cancer survivors in Hong Kong; this pilot tested a 7-week abbreviated version. In this 2-group randomized controlled pilot trial, 34 individuals were assessed for eligibility, and 27 were randomized; 24 completed baseline and were included in the modified intention-to-treat analyses (12 per group). The intervention comprised a progressively supervised group-based tele-exercise intervention transitioning to unsupervised sessions, combined with psychological counseling. Outcomes were guided by the RE-AIM (reach, effectiveness, adoption, implementation, and maintenance) framework, with emphasis on feasibility, acceptability, and preliminary effects.

**Results:**

Recruitment was 79.4% (27/34) and baseline-to-post retention was 100% (24/24), with satisfactory attendance (87.7%) and compliance (85.3%) in the intervention group. Acceptability was high. Preliminary signals of improvement were observed in cardiorespiratory fitness (primary outcome), lower-extremity strength, balance, affected-side shoulder range of motion, and health-related quality of life.

**Conclusions:**

This pilot supports the feasibility and acceptability of tele-exercise for breast cancer rehabilitation and provides preliminary evidence to justify a larger trial with longer follow-up to assess sustained effects and broader applicability.

## Introduction

Globally, breast cancer is the most prevalent cancer in women; in 2022, an estimated 2.3 million women were diagnosed with breast cancer and 670,000 died from the disease [1]. A review further reported that breast cancer incidence and mortality increased by 20% and 14%, respectively, from 2008 to 2012 [2]. Similarly, in Hong Kong, breast cancer is the most common cancer in females. The latest statistics show that breast cancer accounted for 28.9% of all new cancers in females in 2023 (n=5585) [3]. It is also the third leading cause of cancer mortality, following lung and colorectal cancers and accounting for 13.1% of cancer mortality in females in 2024 [3].

Adjuvant therapy (ie, treatment after surgery) is the standard treatment. More than 90% of all patients receive an adjuvant therapy [4]. In Hong Kong, most cases after surgery receive a combined treatment of radiation and chemotherapy. Radiation therapy only usually results in short-term side effects (such as fatigue and skin burns), while those receiving chemotherapy may suffer from both short-term (such as fatigue and gastrointestinal symptoms) and long-term consequences (such as weight gain and cognitive dysfunctions) [5-7]. These adverse effects are associated with decreased physical and mental health, decreased health-related quality of life, and increased risk for mortality [2].

Evidence has shown that 70% of newly diagnosed cases are insufficiently active (<150 minutes/week) [8]; >60% of patients reduce physical activity levels postdiagnosis [8], with greater declines observed in those receiving adjuvant radiation therapy with chemotherapy [2]; both insufficient activity and declines in physical activity levels can elevate risks for the side effects of adjuvant treatments [8]. A large body of research has suggested exercise-induced effects in ameliorating cancer and treatment-related symptoms and improving health. Two recent Cochrane reviews have further suggested that exercise can elicit beneficial effects on fatigue, cardiopulmonary fitness, muscular strength, depression, and health-related quality of life with moderate-quality evidence (indicating further studies may change the estimate), while evidence in cognitive function, lymphedema, weight, anxiety, and physical activity behaviors is low- or very low-quality (indicating the estimate is likely or very likely to change with further studies) [2,4,5]. Supervised exercise consisting of both aerobic and resistance training is consistently suggested for breast cancer patients due to its greater effects than unsupervised modes [2,4,5]. However, its sustainability of both exercise habits and beneficial effects after cessation is mixed and critical [2,4,5].

Along with advances in technology, online-based interventions have been emerging in recent years. Particularly, tele-exercise is a novel approach to the delivery of exercise training using information and communication technologies such as the internet, computers, mobile devices, software, and smartphone apps, and allows participants to exercise at home with supervision [9]. The common barriers against exercise in breast cancer survivors include tiredness, transportation, and lack of time [10]. Thus, the home-based tele-exercise approach will help break down these barriers and improve participation and retention of participants. Compared to the traditional face-to-face mode, this approach can also reduce health care burden and costs from both health care providers and patients [9]. Some evidence, though sparse, has suggested that the tele-exercise approach is as effective as the conventional face-to-face exercise to manage disease and promote health [11]. Home-based interventions with minimal exercise equipment were effective and viable alternatives to equipment-intensive regimes [11].

Collectively, exercise can help improve health and reduce treatment-related symptoms among breast cancer survivors. However, strategies are needed to support the sustainability of exercise after supervised programs end, and tele-exercise is a promising approach that warrants evaluation. To address these gaps, we developed a theory-informed tele-exercise program integrating behavior change techniques (BCTs) and a progressive transition from supervised to unsupervised delivery to support habit formation. This paper reports a 7-week pilot study, with the primary objective of assessing acceptability and feasibility and secondary objectives of obtaining preliminary estimates (between-group differences and effect sizes) to inform the design and sample size of the 12-week trial.

## Methods

### Study Design and Setting

This 2-group pilot randomized controlled trial (RCT) of a tele-exercise program was conducted in Hong Kong in 2024. About 27 participants were randomized; 24 completed the baseline assessment and were included in the modified intention-to-treat (mITT) analyses. The study was reported in accordance with the CONSORT (Consolidated Standards of Reporting Trials) extension for randomized pilot and feasibility trials (Checklist 1), and the evaluation was guided by the RE-AIM (reach, effectiveness, adoption, implementation, and maintenance) framework. Outcome assessors and data analysts were blinded to group allocation. A multidisciplinary task force (health care professionals, exercise science and psychology experts, clinical trial specialists, data analysts, coaches, and a local breast cancer survivor association) supported intervention development and delivery.

### Ethical Considerations

Ethical approval was obtained from the Research Ethics Committee of the Hospital Authority of Hong Kong (KC/KE-23‐0122/ER-4). The trial was registered on ClinicalTrials.gov (NCT06382441) and conducted in accordance with the Declaration of Helsinki and ICH-GCP (International Council for Harmonisation of Technical Requirements for Pharmaceuticals for Human Use – Good Clinical Practice). All participants provided written informed consent and were informed of their right to withdraw at any time.

Confidentiality was protected through restricted access to study files, secure storage of paper records in locked cabinets, and encryption of electronic data (including SSL/TLS [Secure Sockets Layer/Transport Layer Security] encryption for any web-based data transfer). Personally identifying information and the ID linkage file were stored separately from the analysis dataset, which was deidentified prior to analysis and reporting. Study documents are retained for 5 years after study completion in line with hospital authority/hospital policies and are securely destroyed thereafter. No identifiable participant information is presented in this paper or multimedia appendices.

### Participants and Recruitment

Breast cancer survivors in the adjuvant-treatment period (within 6 weeks after completion of chemotherapy, with radiotherapy as per usual care) were recruited from the Breast Centre at Kwong Wah Hospital, Hong Kong, between November 2023 and April 2024. Inclusion criteria included (1) females aged 40‐64 years, (2) within 6 weeks after completion of chemotherapy, (3) without severe anemia, (4) without cancer metastasis, (5) able to read and communicate in Cantonese or Mandarin, and (6) smartphone users. Those with any medical, physical, or psychological conditions that may limit participation were excluded from the study, such as uncontrolled severe cardiovascular disease, schizophrenia, and severe neurological dysfunction. Eligibility was assessed by a case manager of breast cancer (a nurse consultant), consulting with a surgeon or a physiotherapist if needed.

Previous studies have suggested that 10% of the estimated sample size of the main study (n=130) is recommended for a simple-armed pilot (ie, intervention only). Given that a control group was included, the estimated sample size for the pilot was 24. Several effective strategies for participant recruitment and retention suggested by previous studies were adopted, including emphasizing study benefits to participants (eg, free assessments of the rehabilitation process on physiological and psychological functions) [12].

### Randomization and Allocation Concealment

Participants were randomized in a 1:1 ratio to the intervention or control group using permuted block randomization with randomly varying block sizes (4, 6, or 8). The randomization schedule was computer-generated prior to recruitment by an independent staff member not involved in enrollment, intervention delivery, or outcome assessment.

Allocation concealment was implemented using sequentially numbered, opaque, sealed envelopes (SNOSE) prepared in advance and stored by independent research staff. After eligibility confirmation and written consent, the next envelope was opened to reveal the group assignment. Baseline assessments were scheduled immediately after randomization; however, 3 participants withdrew after allocation and before completing the baseline assessment. Outcome assessors and data analysts remained blinded to group allocation at both baseline and postintervention assessments. Intervention coaches were not blinded because they delivered the program, but they were not involved in outcome assessments or data analysis. Coaches did not have access to the randomization sequence/allocation list or outcome data beyond participant contact details and scheduling information required for intervention delivery.

### Intervention Group (Group A)

The tele-exercise (Table S1 in Multimedia Appendix 1), consisting of both aerobic and resistance exercises with intensity progressively increasing to a moderate level, was delivered to patients in the intervention group for 7 weeks (150 minutes per week). Delivery mode was transitioned gradually from supervised in week 1 (3 sessions/week, 30‐50 minutes/session) to mixed mode with 1 supervised session and 2 unsupervised sessions in week 7 (Table S2 in Multimedia Appendix 1).

**Figure 1.**
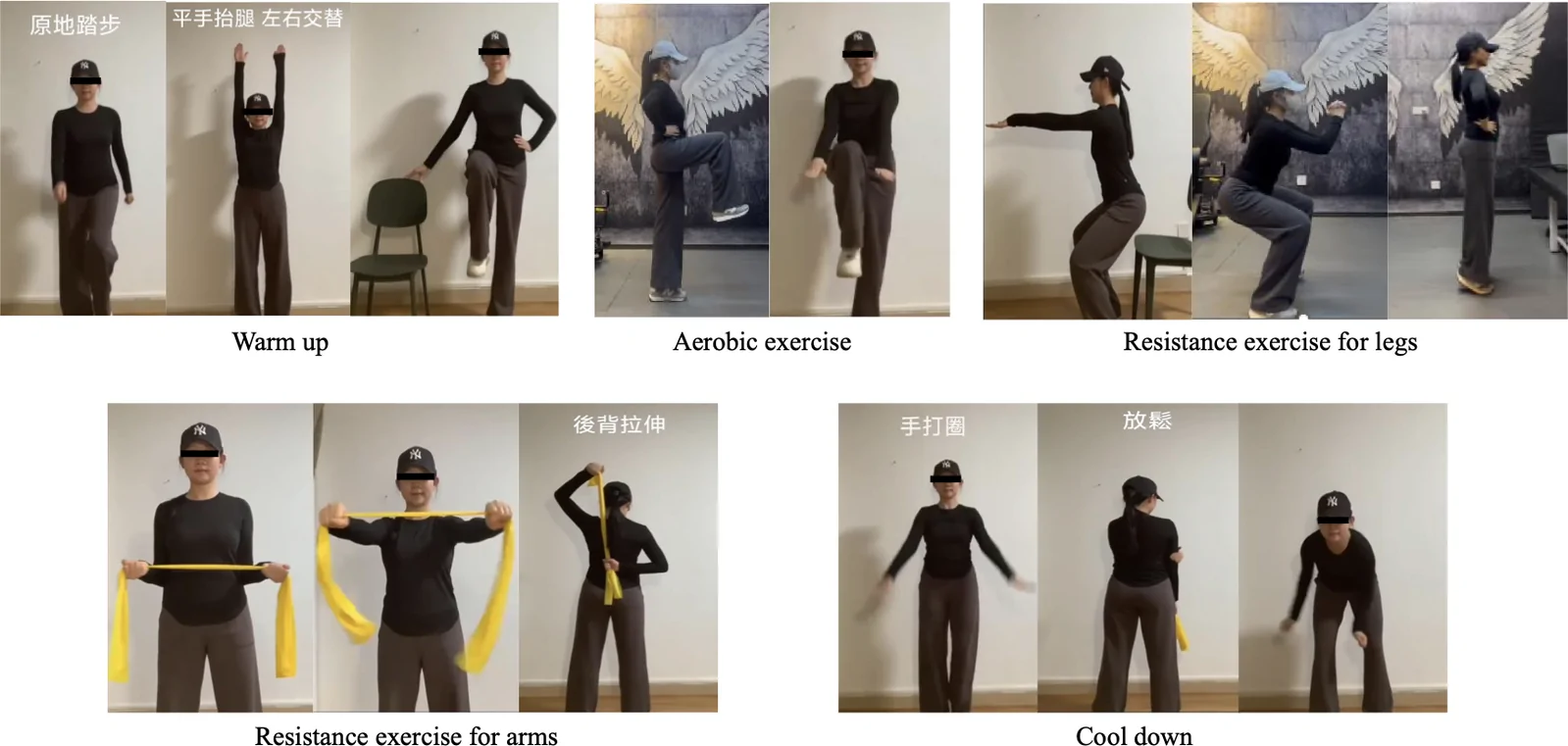
Exercises of the tele-exercise for the pilot study.

Twelve participants formed two groups (6 participants per group) according to their individual exercise intensity levels determined prior to the intervention for each participant. Each group received a total of 15 online supervised training sessions. Each session consisted of warm-up, aerobic exercise, resistance exercise, stretching, and cool-down, with the exercise period progressively increasing from 30 minutes to 50 minutes and exercise intensity progressively increasing from low to moderate levels. Lighter intensity was adopted when there was a need (eg, for those with severe conditions). Target heart rate during exercise was monitored by a wearable activity monitor watch (Mi Band 5, Xiaomi Corporation) to ensure the real exercise intensity (%HRR*,* heart rate reserve) fulfilled the individualized requirements. Cameras were requested to be turned on all the time. Sessions were delivered by a certified fitness instructor (coach) and an assistant coach. Both were coinvestigators of the study. The lead coach held a Certificate in Fitness Instruction (Hong Kong Baptist University), and the assistant coach held a master’s degree in Sport and Leisure Management (Hong Kong Baptist University). For quality control, YG (the principal investigator) participated in the first 3 sessions in week 1 and attended 1 session per week in weeks 2‐4 without prior notification. Figure 1 illustrates some exercise examples. Starting from week 4, unsupervised sessions were introduced, with one session in weeks 4 and 5 and two sessions in weeks 6 and 7. Participants were asked to complete these sessions at home by watching pre-made videos for them. They were also asked to record their exercise (eg, session duration) in an exercise log and to record heart rate using the Mi Band 5.

In order to promote habit formation of exercise, in each supervised session, we included effective behavior counseling elements. These elements were guided by an integrated theoretical framework targeting motivational, volitional, and habit-formation processes, drawing on dual-process accounts of behavior (reflective vs automatic processes) and habit formation theory to support both behavior change and maintenance. The counseling content was developed based on established psychological theories and BCTs used in our previous studies [12-15] and tailored to unique facilitators and barriers to exercise in breast cancer survivors. Seven psychological counseling sessions (30 minutes per session) were delivered by the same coach and assistant from the tele-exercise sessions at a frequency of once per week for 7 weeks. The lead coach had previous experience as a research assistant in a tele-exercise intervention for patients recovering from COVID-19, co-led by the principal investigator (YG), where she supported the delivery of educational workshops. For the present trial, the coach completed three 1-hour training sessions focused on counseling content and techniques tailored to breast cancer survivors and undertook several practice sessions to further refine counseling delivery.

The psychological strategies aimed at changing motivation, self-regulation skills, and habit formation of exercise. Motivational content targeted reflective motivation (eg, strengthening intentions and outcome expectancies), volitional content targeted self-regulation and action control (eg, planning and self-monitoring), and habit-formation content targeted automaticity by establishing stable contextual cues and repetition. For example, the participants were advised on how to work toward their goals and maintain motivation in the face of barriers (motivational strategy); they were encouraged to set outcome-specific goals and self-monitor their progress toward the goals by monitoring how much time they spent in exercise (self-regulation strategy); and they were asked to recognize and identify situational, contextual, and time-based cues that could prompt them to be active (habit development strategy). Table S3 in Multimedia Appendix 1 details the topics, theoretical strategies, and BCTs involved in each session, and Multimedia Appendix 2 provides the detailed content of session 1 (Chinese only)*.*

On the day of baseline measurements, participants in group A received a face-to-face briefing on installing and using Zoom and on intervention procedures. Additional support was provided before, during, and after the intervention via a hotline and social media (eg, WhatsApp). Participants were asked not to share intervention-related materials (including videos) with other patients to minimize potential contamination.

Safety and potential adverse effects of intervention: It is critical to monitor and record harm or unintended consequences in exercise interventions for breast cancer survivors in order to ensure that the interventions are safe and appropriate. In the intervention arm, a pulse oximeter (Innovo Premium Fingertip Pulse Oximeter) was used to monitor oxygen saturation in the initial exercise sessions to avoid exercise-induced oxyhemoglobin desaturation [16]. Participants with lymphedema wore a compression sleeve during exercise. Possible adverse effects, such as lymphedema, fracture, and other arm/shoulder symptoms, suggested by previous studies were monitored and remedied promptly via diverse means (eg, to inform participants of the possible adverse consequences in advance and provide feasible and practical preventive strategies to them) [16,17]. According to the American College of Sports Medicine’s guidelines for exercise, the tele-exercise was stopped immediately in the following situations during exercise: (1) deteriorated upper extremity symptoms; (2) chest pain; (3) central nervous system symptoms (eg, dizziness or near syncope); (4) fatigue, shortness of breath, wheezing, muscular cramps, or claudication; (5) heart rate during exercise ≥90% HRR (the upper limit of vigorous intensity); (6) an absolute decrease in oxygen saturation SpO2≥5% or SpO2≤80% [16].

After the intervention, all participants in the intervention group were interviewed regarding acceptability and feasibility. Secondary health outcomes were measured among all participants before and after the intervention to investigate preliminary effects of the study and to calculate the sample size of the main study.

### Control Group (Group B)

Participants in the control group received 7 educational essays on exercise and health (1 essay per week for 7 weeks). All participants in both groups continued to receive usual care from the study hospital during the study period. Multimedia Appendix 3 provides an example essay (Chinese only)*.*

### Outcomes and Measurements

The pilot was assessed following the RE-AIM framework. Both quantitative and qualitative data were collected via semistructured, face-to-face interviews [18]. The interview included a structured 14-item acceptability and feasibility questionnaire, with each item rated on a 5-point Likert scale (“1=strongly disagree” to “5=strongly agree”). For each item, participants were also invited to elaborate through an open-ended follow-up prompt to explain their rating and provide additional comments.

The structured Likert-scale responses were analyzed quantitatively to summarize acceptability and feasibility across domains, including the individualized exercise design, exercise-intensity progression, session frequency/duration, perceived difficulty of the online guided exercises, scheduling and staffing arrangements, transition to independent activity, perceived usefulness and safety, overall satisfaction, willingness to recommend the intervention, and acceptability of the assessment procedures. The open-ended responses were used to complement and contextualize the quantitative findings.

Given the pilot nature of the study and the supportive role of the qualitative data, we conducted a descriptive content summary. One researcher (SY) reviewed the written interview notes, extracted relevant text, and organized comments into broad categories guided by the interview domains (eg, perceived benefits, barriers, facilitators, and recommendations). The qualitative summaries were used to support the interpretation of the quantitative results rather than to generate formal qualitative themes. No qualitative analysis software was used; data were managed in Microsoft Word. Table 1 details the indicators of acceptability and feasibility.

**Table 1.** Description of indicators of acceptability and feasibility.

Measure	Description of measurement
Acceptability	Participant-rated acceptability, satisfaction, enjoyment, perceptions of safety, difficulty, and tolerability of treatment (using a 5-point Likert questionnaire), with optional open-ended comments; adverse events were monitored throughout the intervention.
Recruitment rate	The proportion of eligible participants who consented and were randomized. $\frac{No.\;of\;participants\;randomized}{No.\;of\;eligible\;participants}\times \;100\%$
Retention rate	The proportion of participants who completed the baseline assessment and remained throughout the entire treatment period. $\frac{No.\;of\;participants\;at\;treatment\;end}{No.\;of\;participants\;at\;baseline}\times \;100\%$
Attendance rate	The proportion of total sessions offered to participants to the actual number of sessions participants attended. $\frac{No.\;of\;sessions\;participants\;attended}{No.\;of\;sessions\;offered}\times \;100\%$
Compliance rate	The proportion of intervention sessions in which participants achieved the prescribed exercise intensity (based on meeting the target heart rate reserve (HRR) zone, Table S1 in Multimedia Appendix 1); missed sessions were coded as not compliant. $\frac{No.\;of\;sessions\;meeting\;target\;intensity}{No.\;of\;sessions\;offered}\times \;100\%$
Fidelity	Content, frequency, duration, and coverage as originally intended. Assessed by the deliverer’s adherence to the originally intended treatment plan.

Health outcomes were assessed pre- and postintervention for all participants, including cardiopulmonary fitness (*primary outcome of the main study*), muscular strength, balance, body composition, affected-side shoulder range of motion (ROM), lymphedema, and health-related quality of life. These outcomes were carefully selected based on literature and clinical importance. Table 2 lists the details of the measurements. A self-administered questionnaire was developed to collect personal information, health-related quality of life, physical activity level, and other related factors.

**Table 2.** Description of measurements.

Measure	Description of measurement
Primary outcome of the main study	
Cardiopulmonary fitness	The 6-minute walk test (m) was performed to estimate cardiopulmonary fitness. Participants walked as far as possible in 6 minutes on a floor without a slope and without running. The distance (m) completed was used. The test was taken only once.
Secondary outcomes of the main study	
Muscular strength	Upper extremity muscular strength was estimated with the handgrip test (kg). Participants, while standing, exerted maximum force to squeeze the instrument. Each hand was tested twice, and the best out of the 4 attempts was recorded. Additionally, the higher result for the affected side arm was noted. For participants with both arms affected, the average of the higher results from each arm was used for analysis. Lower extremity muscular strength was estimated with the timed stand test (seconds). Participants were asked to hold their arms crossed at the height of the chest and stand up and down from a sitting position to complete 10 times as fast as possible. The faster (shorter time) from 2 attempts was used.
Balance	Estimated using the single leg stance test (seconds). It is used to estimate static postural and balance control and is commonly used in clinical settings to monitor neurological and musculoskeletal conditions [19].
Body composition	Body composition was assessed using a body composition analyzer (InBody 270). Percent body fat and BMI (kg/m²) were recorded.
Shoulder range of motion (ROM) on affected side	An arm goniometer was used to measure shoulder joint ROM in forward flexion (degrees).
Limb circumference and lymphedema	Arm circumference was measured with a nonstretch tape at styloid and every 10 cm intervals from the ulnar styloid up to 40 cm distally [20]. Lymphedema in the affected arm was defined as a difference of >2 cm in arm circumference at any measurement point compared to its value before surgery.
Health-related quality of life (HRQOL)	Functional Assessment of Cancer Therapy – Breast (FACT-B), a 5-point Likert instrument designed to measure 5 domains of HRQOL in breast cancer patients, including physical, social, emotional, and functional well-being, and a breast cancer subscale. It consists of 37 items, processes sound valid and reliable, and is sensitive to patient progression. The traditional Chinese version was adopted.
Other measurements of the main study	
Height (*cm*)	Measured using a portable stadiometer (Seca 284).
Physical activity level	Self-reported using the International Physical Activity Questionnaire (short version). Weekly time spent on moderate to vigorous intensity physical activity (MVPA, min/week) was calculated.
Personal and medical information	Measured using either a self-administered questionnaire or obtained from clinical records.

### Data Analysis

Quantitative data were summarized descriptively to evaluate the acceptability and feasibility of the tele-exercise. Qualitative open-ended comments were summarized descriptively to contextualize and supplement the quantitative findings. To preliminarily estimate the health effects of the tele-exercise protocol, changes in health outcomes (eg, cardiopulmonary fitness) were compared between the intervention and control groups using the independent-samples *t* test. Analyses followed an mITT approach including all randomized participants with baseline data. Participants who withdrew after randomization but before baseline assessments were excluded; as 3 participants withdrew postallocation (all before baseline), this may introduce selection bias. In the definitive trial, baseline assessments will be completed prior to allocation disclosure (or allocation will be concealed until after baseline) to minimize postrandomization attrition. Given the pilot nature and limited power, analyses were exploratory and focused on estimation. We report mean differences with 95% CIs and effect sizes (Cohen *d*; interpreted as small 0.2, medium 0.5, large 0.8), and treat *P* values as descriptive with no prespecified threshold for statistical significance [21]. No adjustment for multiple comparisons was applied because outcomes were exploratory. The sample size for the main trial was estimated using the repeated measures ANOVA within-between interaction approach (G*Power, Version 3.1.9.7), with parameters of α=.05, β=.8, two groups and three measurement waves. Cohen *d* was converted to Cohen *f* using *f*=*d*/√3 [22].

## Results

Feasibility was assessed by the following aspects.

### Recruitment and Retention

**Figure 2.**
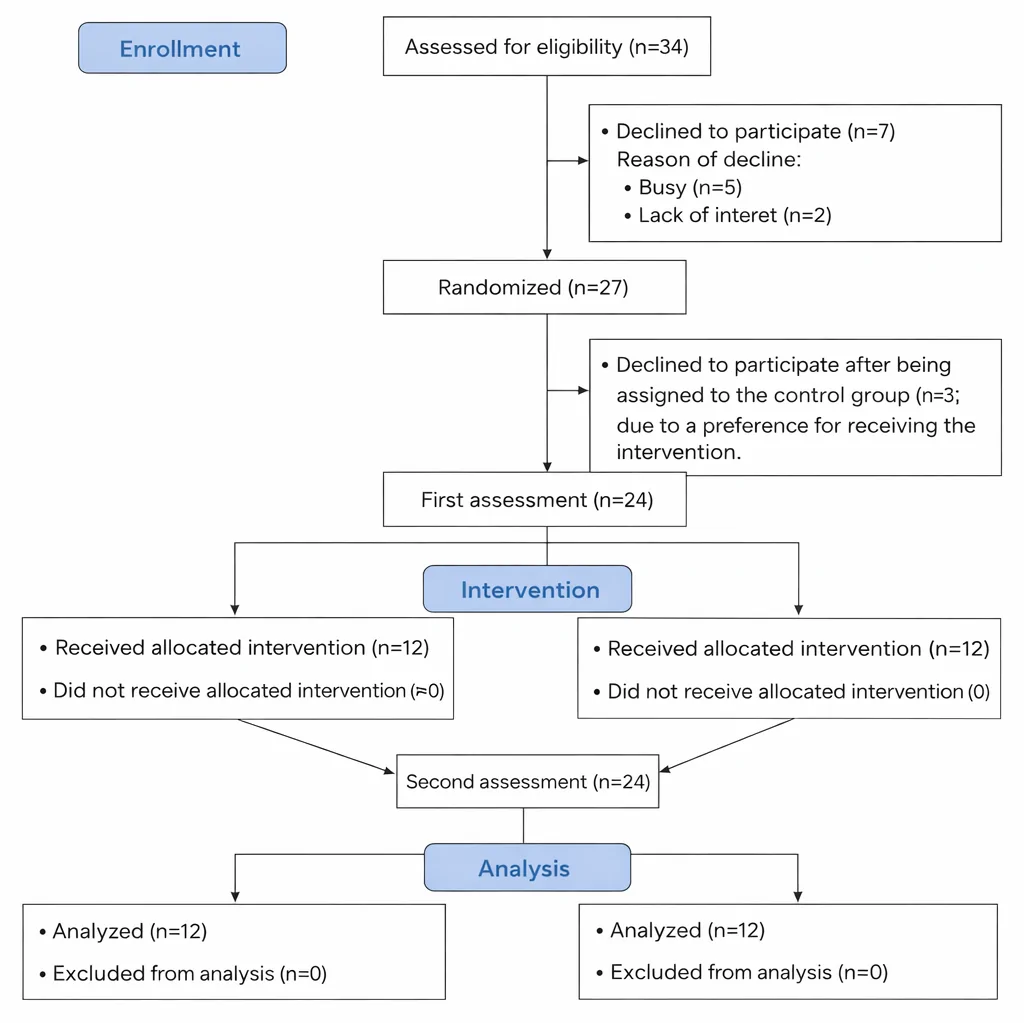
Flowchart of the pilot study.

Of 34 individuals assessed for eligibility, 27 provided consent and were randomized, yielding a recruitment rate of 79.4% (27/34). Of those randomized, 3 participants withdrew after allocation upon learning they had been assigned to the control group and did not complete the baseline assessment; thus, 24 participants completed the baseline assessment and were included in the mITT analyses (12 per group). The remaining 7 eligible individuals declined participation prior to randomization (most commonly due to being busy or lack of interest). All 24 participants who completed baseline also completed the postintervention assessment, resulting in a baseline-to-post retention rate of 100% (24/24). Figure 2 shows the flowchart of the pilot study.

**Figure 3.**
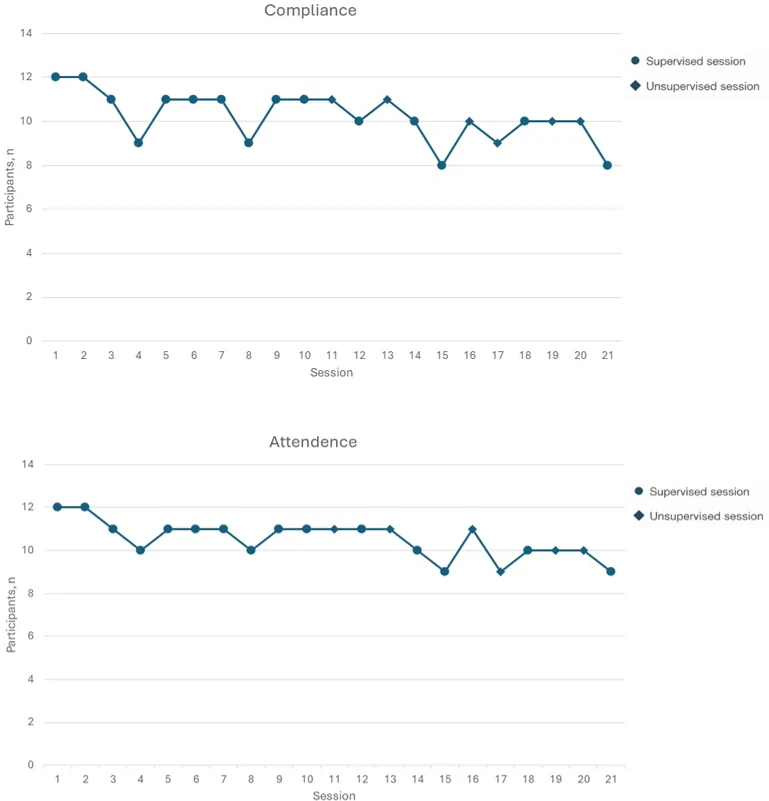
Attendance and compliance rates by session.

Attendance and compliance were summarized separately for supervised and unsupervised sessions (Table 3). Attendance was 88.3% (159/180) for supervised sessions and 86.1% (62/72) for unsupervised sessions. Compliance was 86.1% (155/180) and 83.3% (60/72), respectively. As shown in Figure 3, attendance and compliance were highest at the start (12/12 participants in sessions 1‐2) and then fluctuated modestly, with no obvious decline following the introduction of unsupervised sessions (diamond markers); attendance generally ranged from 9 to 12 participants and compliance from 8 to 12 participants across sessions.

**Table 3.** Attendance and compliance rates of the intervention.

	Overall	Supervised sessions	Unsupervised sessions
Attendance^a^	87.7% (221/252)	88.3% (159/180)	86.1% (62/72)
Compliance^a^	85.3% (215/252)	86.1% (155/180)	83.3% (60/72)

a Rates were calculated using session-level denominators based on scheduled sessions for all participants randomized to the intervention group (n=12): supervised sessions (12×15=180) and unsupervised sessions (12×6=72), totaling 252 sessions. For compliance, missed sessions were counted as noncompliant.

Acceptability of the intervention was assessed through interviews using 14 five-point Likert items among the 12 participants who completed the intervention. All 12 participants completed the acceptability ratings; 5 participants additionally provided optional open-ended written comments. The items covered various aspects, including the individualized approach, exercise intensity, frequency and duration of sessions, difficulty level, time scheduling, staffing arrangement, the transition from supervised to unsupervised delivery modes, usefulness of the psychological elements, safety, perceived effectiveness, satisfaction, willingness to recommend the program to others, and the assessment arrangement. All participants either agreed or strongly agreed with the acceptability of the intervention for half of the items (Table 4). For the remaining items, most responses were positive, with only one neutral response per item. In the optional open-ended comments (n=5), participants reported high satisfaction with the intervention, intention to recommend it to others, and a desire for continuation. One participant described perceived health improvements, including reduced symptoms and improved emotional and mental well-being, and expressed gratitude for the staff’s professionalism and support. Regarding the neutral responses, one participant attributed her rating to missing several sessions due to scheduling conflicts with chemotherapy appointments. Another participant indicated a preference for more sessions per week and therefore rated “frequency and duration” as neutral.

**Table 4.** Acceptability of the intervention from the interview (n=12).

	Strongly agree, n (%)	Agree, n (%)	Neutral, n (%)	Disagree, n (%)	Strongly disagree, n (%)
Individualized approach	9 (75.0)	3 (25.0)	0 (0.0)	0 (0.0)	0 (0.0)
Intensity of exercise	11 (91.7)	1 (8.3)	0 (0.0)	0 (0.0)	0 (0.0)
Frequency and duration	9 (75.0)	2 (16.7)	1 (8.3)	0 (0.0)	0 (0.0)
Difficulty	10 (83.4)	1 (8.3)	1 (8.3)	0 (0.0)	0 (0.0)
Time arrangement	7 (58.4)	4 (33.3)	1 (8.3)	0 (0.0)	0 (0.0)
Staffing arrangement	9 (75.0)	2 (16.7)	1 (8.3)	0 (0.0)	0 (0.0)
Progressively transitioning from supervised to unsupervised modes	11 (91.7)	1 (8.3)	0 (0.0)	0 (0.0)	0 (0.0)
Usefulness of psychological approach	10 (83.3)	2 (16.7)	0 (0.0)	0 (0.0)	0 (0.0)
Safety	11 (91.7)	1 (8.3)	0 (0.0)	0 (0.0)	0 (0.0)
Effectiveness	10 (83.4)	1 (8.3)	1 (8.3)	0 (0.0)	0 (0.0)
Satisfaction	9 (75.0)	2 (16.7)	1 (8.3)	0 (0.0)	0 (0.0)
Recommendation to others	9 (75.0)	2 (16.7)	1 (8.3)	0 (0.0)	0 (0.0)
Arrangement of assessment				0 (0.0)	0 (0.0)
Questionnaire	10 (83.3)	2 (16.7)	0 (0.0)	0 (0.0)	0 (0.0)
Objective measurements	10 (83.3)	2 (16.7)	0 (0.0)	0 (0.0)	0 (0.0)

Fidelity of the intervention was evaluated in terms of content, frequency, duration, and coverage of the intervention.

- Content: All key components of the sessions were covered with minor deviations due to individual participant needs.
- Frequency: All planned supervised exercise and counseling sessions were delivered as scheduled (dose delivered), while participants attended 87.7% of scheduled sessions (dose received).
- Duration: The recorded durations of the sessions were within the expected range of 90-95% of the time. A few sessions lasted longer than the planned duration due to participant fatigue.
- Coverage: All participants in the intervention group who completed baseline (12/12) completed the postintervention assessment, resulting in a 100% coverage rate. There were no reports of intervention materials being shared with the control group, ensuring the integrity of the study conditions.

The results indicated that the intervention was implemented with high fidelity to the original protocol.

- Safety and adverse events: No adverse events, including no serious adverse events or exercise-related adverse events, were reported during the intervention period.

### Health Outcomes

#### Characteristics of the Participants at Baseline

Table 5 shows the characteristics and the first assessment’s results of the 24 participants. The average age of the participants was 57.54 (SD 8.37) years, most were married/cohabitating, and most obtained a secondary education level. The outcome assessment encompassed cardiopulmonary fitness (6-min walk test, *primary outcome*), muscular strength (assessed by handgrip test and timed stand test for upper and lower extremities, respectively), balance (single leg stance test), body composition (percent body fat and BMI), shoulder ROM on the affected side, and health-related quality of life (as indicated by the FACT-B [Functional Assessment of Cancer Therapy – Breast] Trial Outcome Index, FACT-G [Functional Assessment of Cancer Therapy – General] total score, and FACT-B total score). Baseline characteristics were generally comparable between groups, although some numerical differences were observed in handgrip strength.

**Table 5.** Baseline characteristics of the participants.

Characteristics	Total (n=24)	Intervention (n=12)	Control (n=12)
Age (years), mean (SD)	57.54 (8.37)	58.58 (8.14)	56.50 (8.83)
Marital status, n (%)			
Married/cohabitating	14 (58.3)	6 (50.0)	8 (66.7)
Divorced/separated/widowed	5 (20.8)	5 (41.7)	0 (0.0)
Others	5 (20.8)	1 (8.3)	4 (33.3)
Education level, n (%)			
Primary	9 (37.5)	6 (50.0)	3 (25.0)
Secondary	12 (50.0)	6 (50.0)	6 (50.0)
Postsecondary	3 (12.6)	0 (0.0)	3 (25.0)
Height (cm), mean (SD)	155.52 (7.83)	154.88 (7.15)	156.17 (8.72)
Weight (kg), mean (SD)	61.56 (13.18)	62.47 (13.35)	60.65 (13.52)
MVPA^a^ (min), mean (SD)	46.25 (77.67)	51.67 (97.50)	40.83 (55.18)
6-min walk test (m), mean (SD)	508.54 (76.00)	497.83 (83.33)	519.25 (69.87)
Handgrip test (kg*,* best), mean (SD)	22.48 (5.18)	25.21 (4.93)	19.75 (3.93)
Handgrip test (kg*,* affected side)^b^, mean (SD)	20.83 (5.64)	24.00 (5.22)	17.67 (4.17)
Timed stand test (seconds), mean (SD)	17.57 (4.47)	17.38 (4.07)	17.79 (5.06)
Single leg stance test (seconds), mean (SD)	39.39 (24.19)	36.97 (22.80)	41.81 (26.28)
Percent body fat (%), mean (SD)	34.47 (6.95)	35.57 (6.23)	33.37 (7.72)
BMI (kg/m^2^), mean (SD)	25.33 (4.34)	25.85 (3.84)	24.81 (4.91)
Shoulder ROM^c^ (degree*,* affected side)^b^, mean (SD)	165.00 (16.88)	169.58 (10.10)	160.42 (21.16)
Averaged arm circumference (cm*,* affected side)^b^, mean (SD)	23.33 (2.03)	23.95 (1.72)	22.88 (1.66)
FACT-B Trial Outcome Index^d^, mean (SD)	53.50 (11.28)	53.17 (9.92)	53.83 (12.95)
FACT-G total score^e^, mean (SD)	65.31 (14.57)	63.00 (12.98)	67.61 (16.24)
FACT-B total score^f^, mean (SD)	86.81 (18.83)	84.58 (15.75)	89.03 (21.96)

a MVPA: moderate to vigorous intensity physical activity.

b Calculated and used for these cases.

c ROM: range of motion.

d Score range: 0‐96. FACT-B: Functional Assessment of Cancer Therapy – Breast.

e Score range: 0‐108. FACT-G: Functional Assessment of Cancer Therapy – General.

f Score range: 0‐148. FACT-B: Functional Assessment of Cancer Therapy – Breast.

#### Breast Cancer History and Comorbidities

All participants were diagnosed within 1 year prior to the baseline measurement; laterality was right in 11, left in 10, and bilateral in 3. All invasive tumors were invasive ductal carcinoma, mainly grade 2 or 3. Median invasive tumor size was 18 mm (range: 2‐65 mm). Local therapy comprised mastectomy in 22 and lumpectomy in 2; axillary surgery was dissection in 14 and sentinel node biopsy in 10; 2 underwent reconstruction. All had received chemotherapy, and 66.7% had received radiotherapy, mostly to the chest wall and regional nodes. Eight were premenopausal, 15 postmenopausal, and 1 had a first-degree family history; all were alive at last follow-up.

Of the 24 participants, 11 (45.8%) had no comorbidities, 5 (20.8%) had one, 3 (12.5%) had two, 3 (8.3% each) had three or four, and 1 (4.2%) had five comorbidities. Hypertension and hyperlipidemia were each present in 6 (25% each); obesity in 5 (20.8%); diabetes in 4 (16.7%); chronic kidney disease and ischemic heart disease in 2 each (8.3% each); and chronic hepatitis, depression with anxiety, obstructive sleep apnea, anemia, and coronary artery disease in 1 each (4.2% each).

Table 6 presents exploratory between-group comparisons of pre-post change scores. Consistent with the pilot nature of this trial, analyses focused on estimating between-group differences and associated uncertainty rather than formal hypothesis testing for effectiveness. Overall, the direction of effects tended to favor the intervention for the 6-minute walk test (6MWT), timed stand test, single leg stance, affected-side shoulder ROM, and health-related quality-of-life outcomes, indicating outcomes that may be promising to prioritize in a future definitive trial.

**Table 6.** Independent-samples *t* test for changes in outcomes (*post minus pre*)^a^.

Outcomes	Intervention (n=12)	Control (n=12)	Between-group difference in change (*post minus pre*)				
	Mean (SD)	Mean (SD)	Mean differences	95% CI	*P* value	Cohen *d*	Effect size level^b^
6-min walk test (m*,* primary outcome)	39.17 (26.19)	24.25 (19.08)	14.92	−4.48 to 34.31	.13	0.651	Medium
Handgrip test (kg*,* best)	1.08 (1.94)	0.08 (1.82)	1.00	−0.59 to 2.59	.21	0.532	Medium
Handgrip test (kg*,* affected side)^c^	0.71 (1.78)	0.17 (1.86)	0.54	−1.00 to 2.08	.47	0.298	Small
Timed stand test (seconds)	−2.52 (2.26)	−1.08 (2.40)	−1.44	−3.42 to 0.53	.14	−0.619	Medium
Single leg stance test (seconds)	11.72 (20.01)	3.04 (17.99)	8.69	−7.42 to 24.80	.28	0.457	Small
Percent body fat (%)	−1.19 (1.79)	−0.58 (1.24)	-0.62	−1.92 to 0.69	.23	−0.401	Small
BMI (kg/m^2^)	−0.58 (0.70)	−0.28 (0.42)	-0.30	−0.79 to 0.19	.21	−0.522	Medium
Shoulder ROM^d^ (degree*,* affected side)^c^	6.25 (11.31)	−0.83 (10.62)	7.08	−2.21 to 16.37	.13	0.646	Medium
Averaged arm circumference (cm*,* affected side)^c^	−1.06 (0.80)	−0.66 (0.91)	−0.40	−1.13 to 0.32	.26	−0.470	Small
FACT-B Trial Outcome Index^e^	11.00 (14.18)	1.67 (10.63)	9.33	−1.28 to 19.94	.08	0.745	Medium
FACT-G total score^f^	9.26 (14.45)	−2.28 (13.14)	11.54	−0.15 to 23.24	.05	0.836	Large
FACT-B total score^g^	14.26 (18.68)	−0.78 (17.60)	15.04	−0.33 to 30.41	.06	0.829	Large

a Between-group comparisons are exploratory; the study was not powered for effectiveness testing. *P* values are descriptive.

b Level: cutoff points of 0.2, 0.5, and 0.8 were used to classify effect sizes into small, medium, and large levels.

c Three cases (1 in intervention, 2 in control) had both sides affected by surgery, and then the average was calculated and used for these cases.

d ROM: range of motion.

e Score range: 0-96. FACT-B: Functional Assessment of Cancer Therapy – Breast.

f Score range: 0-108. FACT-G: Functional Assessment of Cancer Therapy – General.

g Score range: 0-148. FACT-B: Functional Assessment of Cancer Therapy – Breast.

Absolute effect sizes ranged from 0.298 (handgrip test on the affected side) to 0.836 (FACT-G total score), with half at a medium level, including the primary outcome of the 6-minute walk test (0.651). Based on the effect size of 0.651 for the primary outcome, the required sample size for the main study was calculated to be 102 participants, with 51 participants in each group.

## Discussion

### Main Findings

This pilot RCT evaluated the feasibility, acceptability, and preliminary effects of a 7-week home-based, group-based tele-exercise intervention (with a progressive transition from supervised to unsupervised sessions) plus brief psychological counseling among breast cancer survivors undergoing adjuvant therapy. The intervention demonstrated high feasibility, with a recruitment rate of 79.4% and baseline-to-post retention of 100%, and good engagement, with an overall attendance rate of 87.7% and compliance of 85.3% in the intervention group. The intervention was also highly acceptable, with all participants reporting agreement or strong agreement across key acceptability items. Although the study was not powered for definitive effectiveness testing, between-group differences in change generally favored the intervention across cardiopulmonary fitness (6MWT, *primary outcome*) and showed positive trends in lower-extremity strength, balance, shoulder ROM on the affected side, and several health-related quality-of-life outcomes, warranting confirmation in a fully powered trial.

### Comparison With Previous Studies

The pilot demonstrated strong feasibility, evidenced by high recruitment and retention rates. These rates appear higher than those reported in many exercise and tele-exercise trials among breast cancer survivors [2,4,5,23], which may reflect the study’s pragmatic design and local tailoring (eg, highlighting participant benefits such as free assessments and aligning delivery with local care routines). Engagement was also high: overall attendance was 87.7%, and compliance was 85.3%, comparing favorably with prior exercise interventions [4,5]. The progressive transition from supervised to unsupervised sessions was rated as acceptable (Table 4), and adherence remained broadly comparable between supervised and unsupervised sessions during the intervention period (Table 3 and Figure 3). Figure 3 further suggests that attendance remained stable across the intervention, including after the introduction of unsupervised sessions, which supports the feasibility of reducing supervision without an obvious loss of participation during adjuvant therapy.

In contrast, heart rate–based compliance showed modest session-to-session fluctuations and a slight downward tendency over time. One possible explanation is physiological adaptation: as participants became more familiar with the prescribed workload, the same exercises may have elicited a lower heart-rate response, reducing the likelihood of meeting the original target heart-rate zone despite continued participation [24,25]. This suggests that more dynamic adjustments to the exercise prescription will be necessary in the definitive trial. The brief psychological counseling component may also have helped participants sustain engagement by supporting motivation and self-regulation, although its specific contribution cannot be isolated in this pilot. Tele-exercise delivery at home likely reduced common participation barriers (eg, fatigue, transportation, and time constraints), although longer-term maintenance beyond the intervention period cannot be inferred without follow-up.

Preliminary between-group estimates generally favored the intervention across cardiopulmonary fitness, lower-limb function, balance, shoulder ROM, and health-related quality of life, consistent with meta-analytic evidence in breast cancer survivors [2,4,5,26]. The estimated effect on 6MWT (Cohen *d*=0.65; 95% CI includes 0) suggests the primary outcome is promising to prioritize in a definitive trial. While functional capacity is clinically relevant, this pilot cannot establish clinical effectiveness or downstream outcomes (eg, morbidity/mortality) [27]. From a clinical perspective, the between-group difference in change in 6MWT in this 7-week pilot was approximately 15 m, which is below commonly cited minimal clinically important differences (MCIDs) of 20‐30 m in oncology populations [28]; this might be due to the relatively short duration of the intervention. Therefore, these findings should be interpreted as exploratory; the definitive 12-week trial is needed to determine whether the intervention can achieve clinically meaningful improvements in functional walking capacity.

Changes in physical function were also encouraging. Improvements in the timed stand test and single-leg stance are plausible given the repeated lower-body functional tasks and progressive loading embedded in the sessions. For the timed stand test, lower values indicate faster completion and better performance. These measures are clinically relevant because lower-limb weakness and balance impairment can limit mobility and confidence in daily activities. Shoulder ROM on the affected side also improved, which is notable given the frequency of posttreatment shoulder stiffness; embedding brief mobility work within each session may be a pragmatic way to address this without substantially increasing intervention burden.

Upper-extremity strength (handgrip strength) showed only small between-group differences, which may reflect limited progressive upper-body loading in the 7-week protocol and the need for more targeted upper-body resistance progression training in the main trial. BMI and percent body fat decreased modestly; however, body composition was not a primary target, and large changes were not expected over this short, nondiet intervention.

Quality-of-life measures (FACT-B Trial Outcome Index, FACT-G, and FACT-B total score) also tended to improve in the intervention group. Importantly, the between-group differences in change were approximately 9.33 (FACT-B Trial Outcome Index), 11.54 (FACT-G), and 15.04 (FACT-B total), which exceed commonly cited MCIDs for these scales (FACT-B Trial Outcome Index: 5‐6 points; FACT-G: 5‐6 points; FACT-B total: 7‐8 points) [29]. These preliminary clinically meaningful improvements may reflect both gains in physical function from the combined aerobic-resistance training and the brief behavioral counseling elements (eg, motivation, self-regulation, and habit-formation strategies), which may have helped participants manage barriers and sustain activity. Given the pilot design, findings are exploratory and require confirmation in the definitive trial.

Finally, we did not observe lymphedema exacerbation over the intervention period, and perceived safety ratings were high, which is reassuring and consistent with prior evidence that appropriately prescribed exercise is safe for individuals with breast cancer-related lymphedema [27].

### Strengths and Limitations

This pilot study has several strengths, including a randomized design, blinded outcome assessment, the use of both objective functional outcomes and patient-reported health-related quality of life measures, and a pragmatic tele-exercise intervention that is feasible to deliver during adjuvant therapy.

Several limitations should be considered when interpreting the findings. First, as a pilot study, the trial was designed to evaluate feasibility and acceptability and was not powered to draw definitive conclusions about effectiveness. Accordingly, the small sample (n=24) from a single center in Hong Kong limited the precision of estimates and the generalizability of the findings. Second, the pilot intervention period was limited to 7 weeks, as time and resource constraints did not allow completion of the planned 12-week intervention for the main study. This reduced intervention dose may have limited changes in outcomes and may have led to an underestimation of the potential effectiveness and effect sizes achievable with the intended intervention duration. Attendance and compliance observed over 7 weeks may not be representative of adherence over the planned 12-week intervention, as participation often declines with longer intervention duration. Third, follow-up assessments were not conducted; therefore, the longer-term maintenance of behavior change and durability of outcome improvements could not be evaluated. Fourth, eligibility criteria (language and smartphone use) may limit generalizability; future studies should broaden inclusion and provide support for participants with lower digital access and literacy. Fifth, randomization occurred before baseline assessment. This design may increase postallocation withdrawal due to treatment preference and can introduce selection bias into the analyzed sample. In the future study, baseline assessments will be completed prior to randomization (or, alternatively, randomization will be retained but allocation will be disclosed only after baseline assessment) to minimize postrandomization attrition.

### Implications for the Main Trial and Future Directions

The results of this pilot study provide a foundation for the design and implementation of a larger-scale main study. Based on the observed primary outcome effect size, the estimated sample size for the main study is 102 participants (51 per group). Future research should (1) include postintervention follow-up to evaluate whether benefits persist after supervision is reduced, (2) strengthen fidelity and adherence monitoring and reporting, and (3) consider enhancing upper-body progressive resistance training if upper-limb strength is a priority outcome. In addition, the integration of psychological theories and BCTs in the intervention design may have contributed to habit formation and sustained exercise participation. These elements should be refined and tailored to address the needs and barriers faced by breast cancer survivors across different contexts and settings.

### Conclusions

This pilot RCT demonstrates that a home-based, group-based tele-exercise intervention with brief psychological counseling is feasible and acceptable for breast cancer survivors undergoing adjuvant therapy, with high engagement and no apparent safety concerns. Exploratory between-group estimates for cardiopulmonary fitness, functional performance, affected-side shoulder ROM, and health-related quality of life (effect sizes and 95% CIs) provide parameters to inform the design of a fully powered RCT with longer follow-up to evaluate effectiveness and sustainability.

## Supplementary material

10.2196/79564 Multimedia Appendix 1 Detailed tele-exercise protocol, scheduling, and psychological counseling sessions.

10.2196/79564 Multimedia Appendix 2 Content of session 1 with psychological strategies (as an example) in Chinese.

10.2196/79564 Multimedia Appendix 3 An example essay (in Chinese).

10.2196/79564 Checklist 1 CONSORT checklist.

## Abbreviations

6MWT: 6-minute walk test
BCT: behavior change technique
CONSORT: Consolidated Standards of Reporting Trials
FACT-B: Functional Assessment of Cancer Therapy – Breast
FACT-G: Functional Assessment of Cancer Therapy – General
HRR: heart rate reserve
ICH-GCP: International Council for Harmonisation of Technical Requirements for Pharmaceuticals for Human Use – Good Clinical Practice
MCID: minimal clinically important difference
mITT: modified intention-to-treat
RCT: randomized controlled trial
RE-AIM: reach, effectiveness, adoption, implementation, and maintenance
ROM: range of motion
SNOSE: sequentially numbered, opaque, sealed envelopes
SpO2: oxygen saturation
SSL/TLS: Secure Sockets Layer/Transport Layer Security

## References

[1] (2026). Breast cancer. World Health Organization.

[2] Lahart IM, Metsios GS, Nevill AM, Carmichael AR (2018). Physical activity for women with breast cancer after adjuvant therapy. Cochrane Database Syst Rev.

[3] (2026). Breast cancer. Centre for Health Protection.

[4] Lipsett A, Barrett S, Haruna F, Mustian K, O’Donovan A (2017). The impact of exercise during adjuvant radiotherapy for breast cancer on fatigue and quality of life: a systematic review and meta-analysis. Breast.

[5] Markes M, Brockow T, Resch KL (2006). Exercise for women receiving adjuvant therapy for breast cancer. Cochrane Database Syst Rev.

[6] Martins AD, Brito JP, Oliveira R (2021). Relationship between heart rate variability and functional fitness in breast cancer survivors: a cross-sectional study. Healthcare (Basel).

[7] Arab C, Dias DPM, Barbosa RT de A (2016). Heart rate variability measure in breast cancer patients and survivors: a systematic review. Psychoneuroendocrinology.

[8] Hayes S SK (2014). Is unsupervised exercise following breast cancer safe for all women?. Int J Phys Med Rehabil.

[9] McNamara RJ, Dale M, McKeough ZJ (2019). Innovative strategies to improve the reach and engagement in pulmonary rehabilitation. J Thorac Dis.

[10] Pudkasam S, Polman R, Pitcher M (2018). Physical activity and breast cancer survivors: importance of adherence, motivational interviewing and psychological health. Maturitas.

[11] Jiang Y, Liu F, Guo J (2020). Evaluating an intervention program using WeChat for patients with chronic obstructive pulmonary disease: randomized controlled trial. J Med Internet Res.

[12] Gao Y, Zhong LLD, Quach B (2021). COVID-19 rehabilitation with herbal medicine and cardiorespiratory exercise: protocol for a clinical study. JMIR Res Protoc.

[13] Sun Y, Wang A, Yu S (2020). A blended intervention to promote physical activity, health and work productivity among office employees using intervention mapping: a study protocol for a cluster-randomized controlled trial. BMC Public Health.

[14] Michie S, Richardson M, Johnston M (2013). The behavior change technique taxonomy (v1) of 93 hierarchically clustered techniques: building an international consensus for the reporting of behavior change interventions. Ann Behav Med.

[15] Sun Y, Gao Y, Ou AYT (2026). Effectiveness of a blended intervention to promote physical activity among office employees: randomized controlled trial. J Med Internet Res.

[16] Riebe D, Ehrman JK, Liguori G, Magal M (2018). ACSM’s Guidelines for Exercise Testing and Prescription.

[17] Campbell KL, Winters-Stone KM, Wiskemann J (2019). Exercise guidelines for cancer survivors: consensus statement from international multidisciplinary roundtable. Med Sci Sports Exerc.

[18] Pfledderer CD, von Klinggraeff L, Burkart S, Wolfenden L, Ioannidis JPA, Beets MW (2023). Feasibility indicators in obesity-related behavioral intervention preliminary studies: a historical scoping review. Pilot Feasibility Stud.

[19] Sibley KM, Straus SE, Inness EL, Salbach NM, Jaglal SB (2013). Clinical balance assessment: perceptions of commonly-used standardized measures and current practices among physiotherapists in Ontario, Canada. Implement Sci.

[20] Yuan RZ, Li KP, Wei XL (2021). Effects of free range-of-motion upper limb exercise based on mirror therapy on shoulder function in patients after breast cancer surgery: study protocol for a randomized controlled trial. Trials.

[21] Eldridge SM, Chan CL, Campbell MJ (2016). CONSORT 2010 statement: extension to randomised pilot and feasibility trials. BMJ.

[22] Cohen J (2013). Statistical Power Analysis for the Behavioral Sciences.

[23] Galiano-Castillo N, Cantarero-Villanueva I, Fernández-Lao C (2016). Telehealth system: a randomized controlled trial evaluating the impact of an internet-based exercise intervention on quality of life, pain, muscle strength, and fatigue in breast cancer survivors. Cancer.

[24] American College of Sports Medicine (2017). ACSM’s Exercise Testing and Prescription.

[25] Mytinger M, Nelson RK, Zuhl M (2020). Exercise prescription guidelines for cardiovascular disease patients in the absence of a baseline stress test. J Cardiovasc Dev Dis.

[26] Schmitz KH, Campbell AM, Stuiver MM (2019). Exercise is medicine in oncology: engaging clinicians to help patients move through cancer. CA Cancer J Clin.

[27] Jones LW, Habel LA, Weltzien E (2016). Exercise and risk of cardiovascular events in women with nonmetastatic breast cancer. J Clin Oncol.

[28] Bohannon RW, Crouch R (2017). Minimal clinically important difference for change in 6-minute walk test distance of adults with pathology: a systematic review. J Eval Clin Pract.

[29] Eton DT, Cella D, Yost KJ (2004). A combination of distribution- and anchor-based approaches determined minimally important differences (MIDs) for four endpoints in a breast cancer scale. J Clin Epidemiol.

