# Correction: Effectiveness of a Home-Based and Group-Based Tele-Exercise Program for Breast Cancer Survivors: Pilot Randomized Controlled Trial

**DOI:** 10.2196/114459

**Published:** 2026-11-11

**Authors:** Yang Gao, Yan Sun, Xueying Wang, Oi Kwan Chun, Brigitte Kim Yook Fung, Wai Wun Sara Fung

**Affiliations:** 1 Department of Sports and Health Sciences, Academy of Wellness and Human Development, Faculty of Arts and Social Sciences, Hong Kong Baptist University, AAB 935, Level 9, Academic and Administration Building, Baptist University Road Campus, Hong Kong, China (Hong Kong), 852 3411 7770; 2 Department of Surgery, Kwong Wah Hospital, Hong Kong, China (Hong Kong); 3 Department of Physiotherapy, Kwong Wah Hospital, Hong Kong, China (Hong Kong)

In “Effectiveness of a Home-Based and Group-Based Tele-Exercise Program for Breast Cancer Survivors: Pilot Randomized Controlled Trial” [1], the authors noted two errors.

Affiliation 2 was a duplication of affiliation 1, so it was removed and subsequent affiliations were renumbered accordingly. Affiliation 1 previously appeared as:

Department of Sports and Health Sciences, Academy of Wellness and Human Development, Hong Kong Baptist University, Hong Kong, China (Hong Kong)

This has been amended to add the faculty, as follows:

Department of Sports and Health Sciences, Academy of Wellness and Human Development, Faculty of Arts and Social Sciences, Hong Kong Baptist University, Hong Kong, China (Hong Kong)

All other corresponding author information remains unchanged.

In addition, Figure 3 was originally intended to contain two panels (panel A and panel B). However, due to a figure formatting issue related to system constraints during the production process, only one panel appeared in the final published version of the article, while the second panel was inadvertently omitted.

This correction restores the complete Figure 3 as originally intended by the authors. The revised Figure 3 includes both panel A and panel B. No other content of the manuscript has been changed.

The proposed revised Figure 3 (included here as Figure 1) should appear as follows.

**Figure 1.**
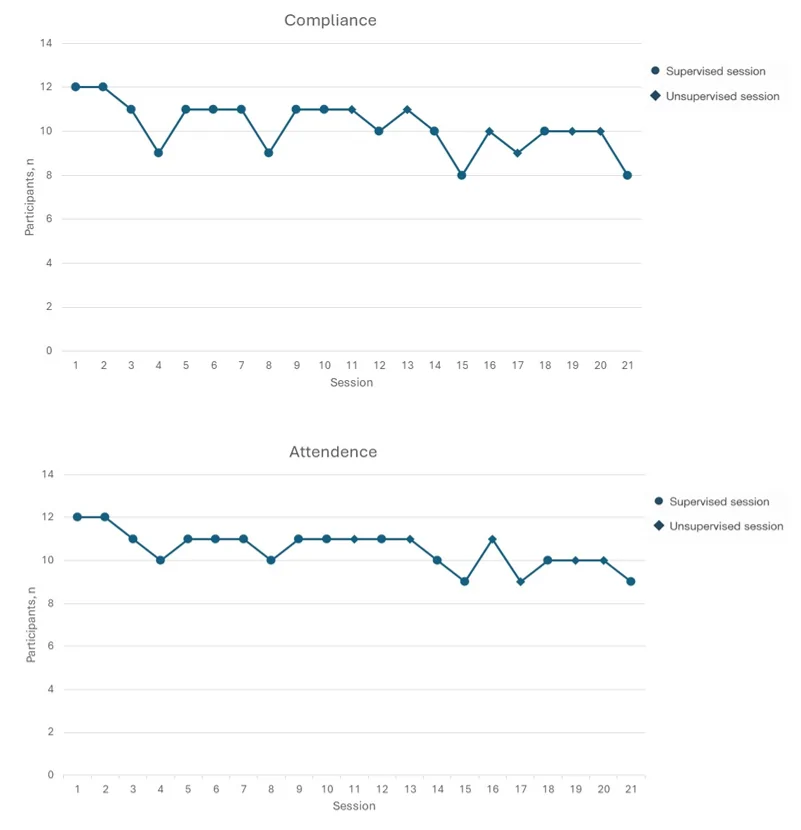
Corrected Figure 3: Attendance and compliance rates by session.

The correction will appear in the online version of the paper on the JMIR Publications website together with the publication of this correction notice. Because this was made after submission to PubMed, PubMed Central, and other full-text repositories, the corrected article has also been resubmitted to those repositories.
