# Supplementary material for: Effectiveness of a Home-Based and Group-Based Tele-Exercise Program for Breast Cancer Survivors: Pilot Randomized Controlled Trial
Source: J Med Internet Res. 2026 Jun 26;28:e79564. doi: 10.2196/79564 (PMC13308909; doi:10.2196/79564)
Supplement: Multimedia Appendix 1 [file jmir-v28-e79564-s001.docx]

**Table S1.** Description of the modified tele-exercise intervention protocol used for the pilot.

|  | Weeks 1-3 (Stage 1) | Weeks 4-7 (Stage 2) |
| --- | --- | --- |
| Dose | 90-150 min/week | 150 min/week |
| Intensity | Light to moderate | Moderate |
| Exercises |  |  |
| *Warm up (3-5 min)* | Stretching, stationary walking | Stretching, stationary walking |
| *Aerobic exercise*  *(10-20 min)* | - Intensity:  30-39%HRR* to 40-49%HRR  - Exercises:  Stationary walking with arm flexion, extension, and circumduction, high knees | - Intensity:  *40-49%HRR* to 50-59%HRR*  Exercises:  Stationary walking with arm flexion, extension, and circumduction, high knees |
| *Resistance exercise*  *(14-25 min)* | - Intensity  30-40% 1-RM**, 8-12 repetitions, 1-2 sets  - Exercises:  Horizontal extension, extension, and lat pull using elastic band, seated leg extensions, heel lifting | - Intensity:  *40-50% 1-RM**, 8-12 repetitions, 2-3 sets*  - Exercises:  Horizontal extension, extension, and lat pull using elastic band, seated leg extensions, heel lifting |
| *Cool down (3-5 min)* | Stretching, stationary walking, massage | Stretching, stationary walking, massage |

*: HRR, heart rate reserve; ** 1-RM, one repetition maximum.

**Table S2.** An example of the time schedule (the first intervention group).

| Week | Monday | Wednesday | Friday |
| --- | --- | --- | --- |
| 1-3 | Supervised | Supervised | Supervised |
| 4 | Supervised | *Unsupervised* | Supervised |
| 5 | *Unsupervised* | Supervised | Supervised |
| 6-7 | *Unsupervised* | *Unsupervised* | Supervised |

**Table S3.** Description of 7 psychological counselling sessions (once per week).

| **Week** | **Topics addressed** | **Theoretical strategy involved/**  ***Personally-tailored feedback*** | **BCTs involved/Name *(code)**** |
| --- | --- | --- | --- |
| 1 | - Exercise in the past week - Exercise goal - Exercise self-efficacy | - Exercise guideline related to main motivation selected *(motivational strategy)* - Feedback on current exercise related to the main motivation *(self-regulation strategy)* - Setting a short-term goal based on self-efficacy level *(self-regulation strategy)* | - Goal setting (behaviour)*(1.1)* - Feedback on behaviour *(2.2)* - Information about health consequences *(5.1)* - Information about emotional consequences *(5.6)* |
| 2 | - Exercise self-monitoring - Habit strength | - Developing habits of exercise *(habit development strategy)* - Exercise progress feedback *(self-regulation strategy)* | - Feedback on behaviour *(2.2)* - Habit formation *(8.3)* - Verbal persuasion of capability *(15.1)* |
| 3 | - Exercise in the past week - Goal setting - Habits & prompts | - Exercise guidance refresher based on the main goal *(self-regulation strategy)* - Exercise progress feedback *(self-regulation strategy)* - Long-term SMART goals *(motivational strategy)* - Feedback on developing habits and noticing prompts *(habit development strategy)* | - Goal setting (behaviour)*(1.1)* - Feedback on behaviour *(2.2)* - Prompts/cues *(7.1)* - Habit formation *(8.3)* - Graded tasks *(8.7)* |
| 4 | - Exercise goals (Short-term) - Action planning reference to habit | - Short-term SMART goals *(motivational strategy)* - Action plan *(self-regulation strategy)* - Action plan referring to behaviour repetition in a stable context *(habit development strategy)* | - Goal setting (behaviour)*(1.1)* - Action planning *(1.4)* - Habit formation *(8.3)* - Graded tasks *(8.7)* |
| 5 | - Exercise in the past week - Coping self-efficacy - Action plan completed | - Boosting confidence and staying motivated based on identified barriers *(motivational strategy)* - Exercise progress feedback *(self-regulation strategy)* - Feedback on developing habits *(habit development strategy)* - Noticing prompts *(habit development strategy)* | - Action planning *(1.4)* - Monitoring of emotional consequences *(5.4)* - Anticipated regret *(5.5)* - Habit formation *(8.3)* - Prompts/cues *(7.1)* |
| 6 | - Exercise in the past week - Confidence to recognise prompts | - Exercise progress feedback *(self-regulation strategy)* - Feedback on developing habits *(habit development strategy)* - Action plan with reference to behaviour repetition in a stable context *(habit development strategy)* | - Action planning *(1.4)* - Prompts/cues *(7.1)* - Habit formation *(8.3)* |
| 7 | - Exercise in the past week - Goal setting - Positive social support | - Exercise progress feedback *(self-regulation strategy)* - Positive influence of others and dyadic plans *(motivational strategy)* - Positive influence of others and dyadic plans *(self-regulation strategy)* - Positive influence of others and dyadic routines *(habit development strategy)* | - Review behaviour goal(s) *(1.5)* - Discrepancy between current behaviour and goal *(1.6)* - Review outcome goal(s) *(1.7)* - Social support (practical) *(3.2)* - Social support (emotional) *(3.3)* - Self-reward *(10.9)* |

*: BCTs, behavioural change techniques; names and codes of BCTs were drawn from the following publication: “Michie S, et al. The Behavior Change Technique Taxonomy (v1) of 93 hierarchically clustered techniques: building an international consensus for the reporting of behavior change interventions, Annals of Behavioral Medicine, 2013;46(1): 81-95. doi: 10.1007/s12160-013-9486-6”.
