# Supplementary material for: Effectiveness of a Home-Based and Group-Based Tele-Exercise Program for Breast Cancer Survivors: Pilot Randomized Controlled Trial
Source: J Med Internet Res. 2026 Jun 26;28:e79564. doi: 10.2196/79564 (PMC13308909; doi:10.2196/79564)
Supplement: Multimedia Appendix 2 [file jmir-v28-e79564-s002.pdf]

Content of session 1 with psychological strategies (as an example)

香港浸會大學
HONG KONG BAPTIST UNIVERSITY

促進運動和健康的心理輔導課程

1

1

課程目的

- 教練個性化指導
- 運動障礙應對
- 目標和計劃制定
- 動機、意志力、遵從性及維持性

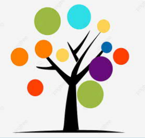

2

第一節課

課程內容：

- 1. 參與本研究的收益（5分鐘）
- 2. 四週的運動康復訓練安排（5-8分鐘）
- 3. 建立個性化健康檔案（3-5分鐘）
- 4. 體力活動回饋（3-5分鐘）
- 5. 設定運動及健康目標（3-5分鐘）

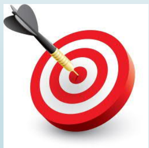

3

1. 參與本研究的收益

1 • 增強身體功能、減輕症狀等

2 • 降低焦慮、抑鬱，提高生活質量等

3 • 提高身體素質，養成健康生活方式

4

2. 四周的遠程運動訓練安排

- 一周三次(30 min)：遠程運動訓練（教練指導）
- 強度的監控
- 強度的遞增
- 一周一次(30 min)：輔導課程

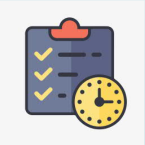

5

3. 個性化健康檔案

康復訓練 — 例如：最大心率，運動強度，參與訓練次數等。

生活方式 — 例如：身體活動、飲食、服藥等。

心理指標 — 例如：心理健康狀態以及社會心理指標等。

生理指標 — 例如：心肺功能、身體成分等。

6

4. 體力活動檔案：鍛煉日誌

7

5. 設定運動訓練目標

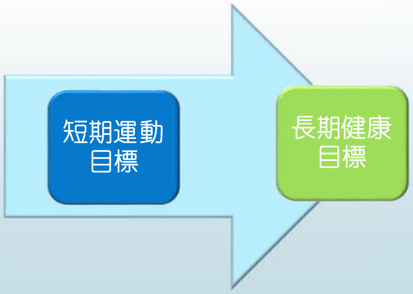

8

謝謝！

有任何疑問，請聯絡教練和學生助手！

香港浸會大學 體育、運動及健康學系

9

第二節課

課程內容：

- 1. 回顧上周體力活動情況（5-8分鐘）
- 2. 评估上周制定的鍛煉目標（3-5分鐘）
- 3. 評估鍛煉的自我效能（5-8分鐘）
- 4. 評估鍛煉的習慣強度（5-8分鐘）
- 5. 建立鍛煉的習慣（5分鐘）
- 6. IMT吸氣肌肉訓練示範（5-8分鐘）

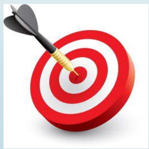

10

1.回顧上周體力活動情況

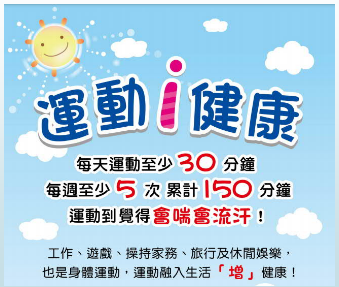

11

2.评估上周體力活動情況

12
