# Supplementary material for: Effectiveness of a Home-Based and Group-Based Tele-Exercise Program for Breast Cancer Survivors: Pilot Randomized Controlled Trial
Source: J Med Internet Res. 2026 Jun 26;28:e79564. doi: 10.2196/79564 (PMC13308909; doi:10.2196/79564)
Supplement: Multimedia Appendix 3 [file jmir-v28-e79564-s003.pdf]

閱讀材料 1

運動與癌症康復

提到做運動，人們總想到有平衡精神狀態、改善機體新陳代謝、增強神經系統機能穩定性或提高免疫力等好處。其實對於患有腫瘤病人來說，體育鍛煉是促進他們康復的法寶。適量運動，可增加病人機體的抵抗力，避免因長期臥床而造成肌肉萎縮、關節僵直或組織器官功能退化。

對於接受腫瘤手術後的病人來說，如恢復良好，無禁忌症，散步、氣功、太極拳、體操或慢跑等，都是一些非常不錯的選擇。而放、化療後的康復者，體育鍛煉也沒有太多的限制，如果身體情況許可，應儘早開始鍛煉，強度可逐步加大。如在嚴重骨髓抑制期(即白細胞降低時)，可暫停運動。

運動對癌症康復者的好處

一、運動能增強人體免疫功能

運動能提高人體製造白細胞的能力。科學研究表明，運動會刺激體內某些激素的分泌，加快骨髓生成白細胞的速度，使白細胞數量增加，存活時間延長，增加吞噬細胞的能力，這樣即使體內出現少量的癌細胞，很快就會被眾多的白細胞圍攻殲滅。另外，人體內的免疫細胞數量和活性也隨運動量的增大而上升，同時呼吸道和消化道黏膜所分泌的抗體數量也會明顯增加。這樣不但可抵禦細菌和病毒的感染，還使人體內特異性和非特異性免疫對腫瘤細胞的殺傷力大大提高。要知道免疫系統是人體內抗擊腫瘤的主要武器，只有擁有這強大的武器，我們才能取得抗癌戰役的最後勝利。

二、運動能調節內分泌水準，尤其是性激素水準

人體內雌激素水準與許多婦科腫瘤如卵巢癌，乳腺癌，子宮內膜癌有顯著的相關性。雌激素在體內新陳代謝中所生成的某些活性產物有利於這些腫瘤進展。而雌激素生成與體內的脂肪量有

關。脂肪越多，這種致癌性物質生成越多。通過有規律的運動可以大大減少體內多餘的脂肪，因此有利於這些腫瘤康復者。

三、運動使血循環加速，阻止癌細胞大量生成，並能將癌細胞處以"死刑"

運動使肌肉產生熱量，劇烈運動甚至可以使體溫上升至 40 度以上。癌細胞對熱的承受能力遠不如正常細胞，較容易被升高的體溫殺死。伴隨著體溫升高，運動還引起大量出汗，汗水可以把體內的一些致癌物質等及時排出體外。另外，運動還使血液循環加速，在這種情況下，體內出現的或是轉移的癌細胞就像急流中的小沙粒一樣，無法在某個內臟器官站穩腳跟，生長發育和轉移擴散。

四、運動使人體吸入更多的氧氣

一般人安靜時每分鐘吸氧量為 4 至 7 升，而運動時可達到 100 升以上。吸氧量的增加，氣體的頻繁交換，可使體內的一些致癌物質排出體外。低氧環境非常有利於腫瘤的生長和轉移，而運動中，尤其是有氧運動過程中，人體吸入的氧氣遠遠多於安靜狀態下，機體內的低氧狀態被糾正，有助於抑制癌細胞的生長。經常進行帶氧運動還可以有效提高抗氧化酶活性，從而不斷清除體內過多的自由基，減少癌症發生的機率。

五、運動能改善人的情緒，消除憂愁煩惱

癌症康復者有部分是由於情緒過於壓抑，精神受到創傷而發病的。而運動可使人心情舒暢，忘卻煩惱。運動時，大腦能產生引起身心愉快的物質"內啡肽"，它可以消除憂愁和煩惱，同時也能消除消極情緒對免疫系統的抑制。

*資料來源：癌症資訊網
https://cancerinformation.com.hk/web
